# Epigenetics, TET proteins, and hypoxia in epithelial-mesenchymal transition and tumorigenesis

**DOI:** 10.7603/s40681-016-0001-9

**Published:** 2016-02-22

**Authors:** Hsiao-Fan Chen, Kou-Juey Wu

**Affiliations:** Research Center for Tumor Medical Science and Graduate Inst. of Cancer Biology, China Medical University, 404 Taichung, Taiwan

**Keywords:** Epigenetics, Hypoxia, TET, DNA methylation, Epithelialmesenchymal Transition, Tumorigenesis

## Abstract

Hypoxia in tumors is primarily a pathophysiologic consequence of structurally and functionally disturbed microcirculation with inadequate supply of oxygen. Tumor hypoxia is strongly associated with tumor propagation, malignant progression, and resistance to therapy. Aberrant epigenetic regulation plays a crucial role in the process of hypoxia-driven malignant progression. Convert of 5-methylcytosine (5mC) to 5-hydroxymethylcytosine (5hmC) by ten-eleven translocation (TET) family enzymes plays important biological functions in embryonic stem cells, development, aging and disease. Recent reports showed that level of 5hmC and TET proteins was altered in various types of cancers. There is a strong correlation between loss of 5hmC and cancer development but research to date indicates that loss of TET activity is associated with the cancer phenotype but it is not clear whether TET proteins function as tumor suppressors or oncogenes. While loss of TET1 and TET2 expression is associated with solid cancers, implying a tumor suppressor role, TET1 exhibits a clear oncogenic role in the context of genomic rearrangements such as in MLL-fusion rearranged leukemia. Interestingly, hypoxia increases global 5hmC levels and upregulates TET1 expression in a HIF1α-dependent manner. Recently, hypoxia-induced TET1 has been demonstrated to play another important role for regulating hypoxia-responsive gene expression and epithelial-mesenchymal transition (EMT) by serving as a transcription co-activator. Furthermore, hypoxia-induced TET1 also regulates glucose metabolism and hypoxia-induced EMT through enhancing the expression of insulin induced gene 1 (INSIG1). The roles and mechanisms of action of 5hmC and TET proteins in ES cell biology and during embryonic development, as well as in cancer biology, will be the main focus in this review.

## 1. Introduction

Insufficient oxygen availability, so-called hypoxia, is a microenvironmental event that plays a critical role in various biological processes including development, metabolism, inflammation, tumor progression and cancer stemness [1, 2]. DNA methylation is one of the epigenetic modifications that plays important roles in numerous cellular processes, including genomic imprinting, X-chromosome inactivation, regulation of gene expression, and maintenance of epigenetic memory [3]. Compared with reversible modifications of histone proteins, DNA methylation was a relatively stable epigenetic modification. The recent finding that teneleven translocation (TET) proteins are 5-methylcytosine (5mC) oxidases has provided the mechanism for the reversal of DNA methylation. In the 1980’s, global hypomethylation was first observed in colorectal cancer cell lines [4]. In addition, genomewide DNA hypomethylation during tumor hypoxia also have been observed [5, 6]. Moreover, several reports demonstrated that hypoxia would enhance TET1 expression and lead to global DNA hypomethylation in different type of cancer [7-9]. Indeed, TET proteins might participate in hypoxia-mediated hypomethylation and lead to enhanced malignancy. Therefore, genome-wide maps of methylation must be systemically established to study the correlation between TET proteins and tumor progression. In this review, we discuss the current knowledge about the mechanism and functions of DNA methylation and TET protein. We also review our current understanding of epigenetic alterations that take place under hypoxia. We then discuss the role of TET proteins and DNA demethylation under hypoxia in epithelial-mesenchymal transition and tumorigenesis.

## 2. Epigenetics and DNA methylation

**Figure 1:**
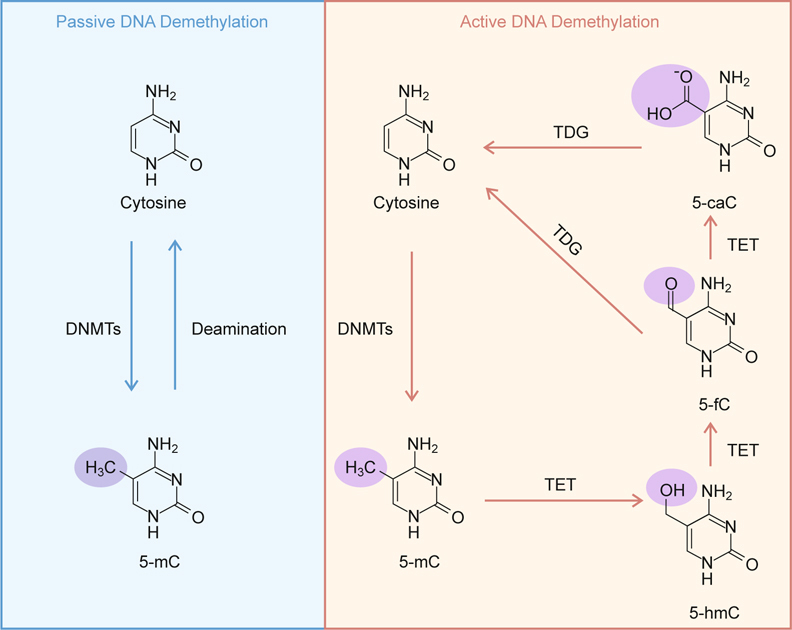

The important role of epigenetic processes in cancer progression and treatment has been emphasized for the past decades. Epigenetic alterations are leading candidates for cancer detection, diagnosis and prognosis [10]. Cancer research in epigenetics in the 1990s was led by a focus on the discoveries and understanding of DNA methylation abnormalities in the 1980s [11]. Abnormal gains of DNA methylation in normally unmethylated gene promoters, leading to transcriptional repression or loss of gene function, are the most widely studied epigenetic alterations in cancer [12]. Moreover, in certain cancers, there is an increasing list of candidate tumor suppressor genes that are silenced by hypermethylation in their promoters [13]. In addition, global hypomethylation has also been implicated in the development and progression of cancer [14]. DNA methylation is established by DNA methyltransferases (DNMTs). The classical model of methylation, which was presented more than 30 years ago, involves two types of DNMTs: maintenance and de novo DNMTs [15, 16]. DNA demethylation involves the removal of the methyl group from 5mC in DNA. This process occurs through two pathways, the passive or active demethylation pathways [17]. Passive mechanisms involve a failure of the repair system to maintain DNA methylation patterns during replication or DNA synthesis and are associated with the dilution of hemimethylated CpG in subsequent replication cycles. Active DNA demethylation involves the replacement of 5mC by cytosine [18, 19]. It has been well recognized that the methyl group of 5mC can also be removed regardless of replication, particularly in the zygotic paternal genome or primordial germ cells. However, the mechanism that causes the active demethylation has remained enigmatic until the function of TET enzymes was identified recently (Figure 1) [20]. Subsequent studies have revealed new visions in the field of DNA methylation and raise many questions about the mechanism and contribution of TET proteins to development and cancer.

## 3. Domain structures of TET family proteins

**Figure 2:**
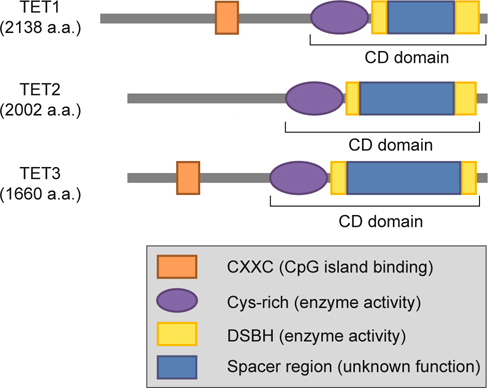

The mammalian TET family contains three members, TET1, TET2 and TET3, all of which share a high degree of homology within their C-terminal catalytic domain - CD domain (Cys-rich and DSBH regions) that belongs to the Cupin-like dioxygenase superfamily and exhibits 2-oxoglutarate (2-OG) - and iron (II)- dependent dioxygenase activity [20, 21]. TET proteins oxidize 5mC into 5-hydroxymethylcytosin (5hmC) through these CD domains and require α-ketoglutarate as a co-substrate for enzymatic activity. Subsequent studies revealed the ability of TET family proteins to further oxidize 5hmC to 5-formylcytosine (5fC) and 5-carboxycytosine (5caC) (Figure 1) [22, 23]. Another mark of TET family proteins is the CXXC zinc-finger domain, which was first identified and defined in DNMT1 [24]. The CXXC domain would be found only in the N-terminus of TET1 and TET3 but not in TET2. Although CXXC domain is known to distinguish between methylated and unmethylated DNA [25], the function of this domain in TET1 and TET3 is largely unknown. It is known that the CXXC domain of TET1 recognizes not only unmodified cytosine but also 5mC and 5hmC, and it favors binding to regions in the genome of high CpG content [26]. In addition to the described functional domains, there is a spacer region that links the two parts of the disconnected DSBH enzymatic domain. This unique spacer region is common to all TET family members, although its length varies. The functional importance of this spacer region is currently unknown (Figure 2). In summary, TET family proteins, through their CD domains, have an enzymatic capability to convert 5mC to 5hmC, 5fC or 5caC depending on the company of co-factors, such as ATP. Furthermore, except CD domain and CXXC domain, TET proteins also contain various additional domains with as yet undefined functions. Its potential regulatory function for modulating TET enzymatic activities deserves future investigation.

## 4. The biological function of TET proteins

Although all TET family members possess 5mC oxidation activity, their expression levels are very different in various cell types and tissues. For example, TET1 and TET2 are highly expressed in mouse ES cells, but TET3 is more enriched in oocytes and onecell zygotes [21]. Evidence suggested that oxidation of 5mC in the paternal genome in fertilized eggs by TET3 initiates DNA demethylation and facilitates the activation of the paternal copy of early embryonic genes [27]. Concomitant with the rapid reduction of TET3 expression at the two-cell stage, the expression of TET1 is rapidly up-regulated at later stage [28, 29]. Result of genome-wide location analyses in mouse ES cells indicated that TET1 and 5hmC are enriched at promoter regions of several pluripotency factors, including *Nanog, Tcl1*, and *Esrrb* [30]. Several genes related to pluripotency are also down-regulated in the double knockdown of TET1 and TET2 of mouse ES cells [31]. Recently, Jaenisch and colleagues generated the TET1-null mouse ES cells and mice to further study the function of TET1 in ES cell maintenance and *in vivo* development, and discovered that TET1 null ES cells maintain their self-renewal ability under mouse ES cell culture conditions *in vitro* and develop normally *in vivo* [32]. Furthermore, TET1 knockout mice and TET2 knockout mice appear to develop normally, and appear healthy through adulthood and are fertile [32, 33]. It is still unclear that the purpose of TET1/TET2 and 5hmC are maintained at high levels in mouse ES cells. As such, the biological role of TET1/TET2 or 5hmC in embryonic development remains unclear. Furthermore, evidence suggests a role for TET enzymes in the generation of pluripotent stem cells (iPSCs) that are phenotypically similar to embryonic stem cells (ESCs) [34]. As shown recently, formation of 5hmC is very important for brain development. In DNA from human brain cortex, the level of 5hmC is about 1% of all cytosines or 20 to 25% of all 5mC bases [35]. TET3 is most highly expressed in the developing mouse brain cortex followed by TET2, while the levels of TET1 are very low in this tissue. An increase in the levels of TET2, TET3 and 5hmC in differentiating neurons corresponds to a decrease in the Polycomb histone H3 lysine 27 (H3K27)-specific methyltransferase EZH2 and loss of H3K27me3 marker at gene promoter. Besides, decreasing the levels of TET2 and TET3 or increasing EZH2 expression results in imperfect neuronal differentiation [36]. Thus, formation of 5hmC promotes neuronal differentiation by modulating the expression of genes most critical in this important developmental transition.

## 5. TET proteins in cancer

In 2011, a chemical labeling technique for determining the genome-wide distribution of 5hmC in human cell lines, as well as in the mouse cerebellum, was developed. A genome-wide study observed that an enrichment of 5hmC in genes linked to hypoxia and angiogenesis [37]. Aberrant DNA methylation is a hallmark of cancer; growing evidence has suggested that an imbalance in TET-mediated DNA demethylation may participate in carcinogenesis. The first reports implicating a role for TET proteins in cancer showed that TET1 is fused to the mixed lineage leukemia (MLL) gene in a case of pediatric AML containing the t(10;11) (q22;q23) [38]. Recent report indicated that TET1 is significantly up-regulated in MLL-rearranged leukemia and is a direct target gene of MLL-fusion proteins. MLL fusions would bind to the promoter region of TET1 to promote its expression directly and result in a global increase of 5hmC. Moreover, together with MLL, TET1 activates the homeobox A9 (Hoxa9)/myeloid ecotropic viral integration 1 (Meis1)/pre-B-cell leukemia homeobox 3 (Pbx3) signaling pathway, which subsequently promotes cell proliferation and inhibits apoptosis/cell differentiation, thereby leading to cell transformation and leukemogenesis [39]. In contrast, many mutations, including nonsense/missense, deletions and frameshift somatic in TET2 were identified in myelodysplastic syndrome (MDS) and other types of leukemia [40, 41]. Some of TET2 mutations could damage the catalytic activity, but many mutations appear unrelated to enzymatic activity [42]. Therefore, the molecular mechanism underlying loss-of-function mutation in TET2 is still unknown and requires further investigation. In addition to AML, TET2 mutations and/or deletions have been observed in other types of tumor, including bladder, breast, kidney, liver, lung and uterine cancers [43]. Since the role of mutated TET proteins in solid cancers has not yet been firmly established, it should be possible to resolve this point by crossing the many available TET-deficient mouse models with the various mouse cancer models available. More importantly, the up-regulation or downregulation of TET gene expression, which is often associated with 5hmC levels, has been observed in numerous solid cancers (Table 1). So far, a direct correlation between TET3 and cancer has not been reported. A possible explanation for this is that TET3 is the most important regulator and critical TET3 mutations or dysregulation might lead to lethality.

**Table 1:** 

Cancer	TET levels	5-hmC level	Correlation with survival	Reference
*MLL*-rearranged leukemia	↑ TET1 ↓ TET2, TET3	–	–	[39]
Invasive ductal carcinoma	↑ TET1, TET3	↑	High TET1, TET3 and 5hmC levels correlate with poor survival	[9]
Breast cancer	↓ TET1, TET2, TET3	↓	Low TET1 levels correlate with poor survival	[86, 87]
Liver cancer	↓ TET1, TET2, TET3	↓	Low 5-hmC levels correlate with poor survival	[86, 88]
Glioma	↓ TET1, TET2, TET3	↓	Low 5-hmC levels correlate with poor survival	[89, 90]
Melanoma	↓ TET1, TET2, TET3	↓	Low 5-hmC levels correlate with poor survival	[91]
Colon cancer	↓ TET1, TET2, TET3	↓	Low TET2 levels correlate with poor survival	[92, 93]
Prostate cancer	↓ TET1	↓	–	[86, 87]
Gastric cancer	↓ TET1	↓	–	[86, 94]
Lung cancer	–	↓	–	[86, 92]
Pancreatic cancer	–	↓	–	[86]

## 6. The role of hypoxia and hypoxia-induced factors in tumor progression

The definition of hypoxia is that a reduction of tissue oxygen tension compares to the normal level. Hypoxia usually occurs during acute and chronic vascular disease, pulmonary disease and cancer [44]. Although hypoxia is toxic to both cancer cells and normal cells, cancer cells undergo genetic and adaptive changes to promote cell survival and even proliferate in a hypoxic environment [45]. Hypoxic tumors are associated with a poor prognosis and resistance to treatments. Well-oxygenated tumor cells have a threefold greater sensitivity to radiation than hypoxic cells [46]. Recently, hypoxia has been identified as an important factor that is correlated with tumor progression including an increasing probability of recurrence, locoregional spread, and distant metastasis [47]. Furthermore, recent studies suggest that tumor hypoxia is associated with malignant biological phenotype such as angiogenesis, migration, invasion and metastasis [48]. The key factor that is involved in adaptive responses to cellular hypoxia is hypoxiainducible factor-1 (HIF-1) and its activity is tightly regulated by the cellular oxygen tension [49, 50]. HIF-1 is a heterodimeric transcription factor that constitutes of a hypoxia-inducible HIF- 1α subunit and a ubiquitously expressed HIF-1β subunit. HIF-1 binds to a 5’-ACGTG-3’ hypoxia-response element (HRE) in the promoter or enhancer of various hypoxia-inducible genes specifically under hypoxia [51]. HIF-1α has been identified as a major regulator of adaptation to hypoxia and implicated in the malignant progression of cancers [52]. Many studies have associated hypoxia and HIF-1α expression with cancer progression. The increased HIF-1α protein level is associated with patient mortality, poor prognosis and treatment resistance in head-and-neck, ovary, colon, breast and lung cancers [53].

## 7. Hypoxia-induced epigenetics

Epigenetic regulation plays an important role in regulating transcriptional changes in hypoxia. Hypoxia in tumor cells display a decrease in the levels of histone acetylation that is associated global transcriptional repression [54]. HDAC expression and activity have been shown to be up-regulated in hypoxia [55]. Hypoxia treatment induces HDAC1 activity and expression. Treatment with TSA, a specific HDAC inhibitor, inhibits hypoxia-induced angiogenesis in the Lewis lung carcinoma model [55]. Some evidences indicate that hypoxia enhances HDACs function and HDACs are implicated in hypoxia-induced metastasis through suppression of hypoxia-responsive tumor suppressor genes [56]. HDAC1 cooperates with HIF-1 downstream target and contribute to suppression of STAT1 [57]. HDAC7 has been found to interact with HIF-1 under hypoxia and increases transcriptional activity of HIF-1 [58]. HDAC4 and 6 also have been found to complex with HIF-1 and associate with HIF-1 stability and transcriptional activity [59]. Under hypoxia, HDAC3, directly activated by HIF-1, would interact with hypoxia-induced WDR5 and recruits the histone methyltransferase (HMT) complex to increase histone H3 lysine 4 (H3K4)-specific HMT activity [60]. Furthermore, treatment with HDAC inhibitor represses HIF-1 induction in response to hypoxia. Hypoxia stimulated proliferation, invasion, migration, and neovascularization are suppressed by treatment with HDAC inhibitors [61]. The O_2_, Fe(II), and α-ketoglutaratedependent jumonji domain (JMJD) histone demethylases are transcriptionally upregulated in hypoxia, and global changes in many histone modifications, especially at the sites of H3 lysine 4, 9, and 36, have also been reported [62-64]. Site-specific changes in histone modifications have been observed at hypoxia-induced genes including CA9, LDHA, and PDK1 [65, 66]. These results support the regulation of hypoxia-responsive gene expression through various histone modifications mediated by histone modifiers.

## 8. DNA methylation status under hypoxia

Recent studies have demonstrated that oxygen levels significantly influence a change in another epigenetic mark, DNA methylation. Hypermethylation of CpG islands in the promoter region can block the expression of HIF-mediated gene expression in hypoxic cells. For example, Bcl-2/adenovirus E1B 19 kDa interacting protein 3 (BNIP3), a regulator of hypoxia-induced cell death, was found to be repressed by DNA methylation in pancreatic, colorectal and gastric cancer [67, 68]. Prolyl hydroxylase (PHD1, PHD2 and PHD3) and factor inhibiting HIF-1 (FIH) had been known as negative regulators of HIF-1 [69]. In the invasive breast carcinoma and colorectal cancer, only PHD3 expression was associated with increased DNA methylation levels in the CpG islands of its promoter [70, 71]. Different from the hypoxia-induced DNA hypermethylation observed at certain loci, hypoxia has been linked to a global reduction in DNA methylation. DNA hypomethylation during hypoxia by examining the amount of 5mC by HPLC was reported in human colorectal and melanoma cell lines [6]. Specifically, CAIX overexpression has been associated with promoter DNA hypomethylation in gastric cancer, and CAIX expression correlates with tumor advancement and metastasis [72]. Moreover, in human hepatoma cell lines, hypoxia induces genomic DNA demethylation through the direct activation of Methionine adenosyltransferase 2A (MAT2A) that maintains the S-adenosylmethionine (SAM)/S-adenosylhomocysteine (SAH) ratio, a critical marker of genomic methylation status [73]. Tumor-associated CpG demethylation leads to increased HIF-1 binding to the HRE and enhanced HIF-1-mediated effects on tumor progression in the HCT116 colon cancer cell line [74]. Human cardiac fibroblast cells exposed to prolonged 1% hypoxia caused a pro-fibrotic state, which was associated with global DNA hypermethylation and increased expression of the DNMTs, such as DNMT1 and DNMT3B [75]. In contrast, down-regulation of DNMT1, DNMT3A and DNMT3B was reported, which contributed to DNA hypomethylation at the proximal promoter region of p16INK4a under hypoxia in human colorectal cancer (HCT116, 379.2) cell lines [76]. These findings support that epigenetic modification, whether global or sitespecific DNA methylation, plays a role in regulation gene expression under hypoxia-induced tumor progression.

## 9. Hypoxia-induced TET proteins upregulation

Hypoxia has also been linked to the HIF-dependent up-regulation of TET1, which catalyzes the hydroxylation of 5mC to 5hmC in tumorigenic N-type neuroblastoma cells exposed to 1% oxygen. TET1 activity essentially leads to DNA demethylation and production of 5hmC, a modification that is associated with active transcription [7]. Recently, Wu *et al*. found that hypoxia would regulate TET1 and TET3 expression through HIF-1α, leading to increased level of global DNA hydroxymethylation that was associated with tumor malignancy in the breast cancer. Furthermore, the results also demonstrated that hypoxia-induced TET1/TET3 proteins made a great contribution to the activation of the TNFα- p38-MAPK pathway in regulating cancer stemness. Histological analysis demonstrated that the levels of 5hmC, TET1 and TET3 were significantly associated with tumor hypoxia, tumor aggressiveness and poor prognosis [9]. TET1 up-regulation leading to global DNA hypomethylation in a HIF-independent manner was also demonstrated in scleroderma fibroblasts [77]. However, the unconventional role of TET proteins in transcription regulation independent of its enzymatic activity has been described in other studies. Many of the genomic regions are inactivated by TET1 through recruitment of the polycomb repressive complex 2 (PRC2), which catalyzes the formation of H3K27me3 and represses gene transcription [78], or it can directly bind to Sin3a histone deacetylase repressive complex to inhibit transcription [79]. Moreover, several groups reported that TET2 and TET3 play an essential part in recruiting O-linked b-N-acetylglucosamine transferase (OGT) to H3K4me3-positive CpG-rich promoters, thus enabling O-GlcNAc modification of histones [80-82]. These findings demonstrate the potential for crosstalk between TET proteins and pathways involved in glucose metabolism. Aberrant glucose metabolism in cancer cells may alter O-GlcNAcylation of TET proteins and therefore affect their stability. Recently, Tsai *et al*. explores the role of TET1 under hypoxia and also proves that TET1 plays another role in serving as a transcriptional coactivator and interacts with HIF-1α and HIF-2α to enhance their transactivation activity independent of its enzymatic activity [8]. It was also reported that TET1 may form a complex with HIF-1/CBP, or with OGT, to facilitate hypoxia-mediated gene expressions [8]. Combined results of RNA sequencing and 5hmC sequencing comparing TET1 knockdown cells under normoxia with those under hypoxia showed that 98 genes were regulated by TET1 and also had increased levels of 5hmC in their promoters. INSIG1 (insulin induced gene 1), a major regulator of cholesterol biosynthesis [83, 84], was inside this list. Furthermore, knockdown of TET1 or INSIG1 diminished a set of hypoxia-induced genes involved in epithelial-mesenchymal transition (EMT), providing a link between lipid metabolism and EMT [8]. Because of the capability of INSIG1 to inhibit cholesterol biosynthesis, it may inhibit lipid synthesis and turn to glucose utilization similar to the role of AMPK that also inhibits cholesterol synthesis [85]. These results demonstrated that the activation of INSIG1 expression through hypoxia-induced TET1 also contributes to Warburg effect, linking hypoxia-regulated TET1/5hmC to metabolism and EMT [8].

## 10. Conclusion and perspectives

The discovery of TET enzymes and the oxidative derivatives of 5mC are a great step for epigenetic research. Studies in the past few years focused on the role of active DNA demethylation in development and disease. Therefore, not surprisingly, changes in TET expression and 5hmC levels have been observed for numerous cancers. However, a deficiency in understanding the mechanism of decreased 5hmC levels and its role in transcriptional control during tumor malignancy still exists. Moreover, TET proteins are sources of 5fC and 5caC. TET protein expression levels and activities as well as the possible readers of all oxidized 5mC derivatives in cancer should be investigated. A potential link between TET proteins and cancer will open a new road for identifying potential therapeutic tools to treat cancer. Moreover, it is now well established that TET proteins are mutated or their expression levels or activities are dysregulated in numerous cancers. Furthermore, investigations are needed to distinguish the catalytic activity-dependent and -independent functions of TET proteins in order to offer new strategies for anti-cancer drug development and cancer therapy.
